# Optimal Screening of Children with Acute Malnutrition Requires a Change in Current WHO Guidelines as MUAC and WHZ Identify Different Patient Groups

**DOI:** 10.1371/journal.pone.0101159

**Published:** 2014-07-01

**Authors:** Arnaud Laillou, Sophonneary Prak, Richard de Groot, Sophie Whitney, Joel Conkle, Lindsey Horton, Sam Oeurn Un, Marjoleine A. Dijkhuizen, Frank T. Wieringa

**Affiliations:** 1 UNICEF, Maternal Child Health and Nutrition section, Phnom Penh, Cambodia; 2 National Nutrition Program, Maternal and Child Health Center, Phnom Penh, Cambodia; 3 Independent consultant, Phnom Penh, Cambodia; 4 World Food Programme, Phnom Penh, Cambodia; 5 World Food Programme, Patumwan, Bangkok, Thailand; 6 Department of Human nutrition, Copenhagen University, Frederiksberg C, Denmark; 7 Institut de Recherche pour le Développement, UMR 204, IRD/Montpellier1/Montpellier2/SupAgro (NUTRIPASS), Montpellier, France; Aga Khan University, Pakistan

## Abstract

**Background:**

Timely treatment of acute malnutrition in children <5 years of age could prevent >500,000 deaths annually. Screening at community level is essential to identify children with malnutrition. Current WHO guidelines for community screening for malnutrition recommend a Mid Upper Arm Circumference (MUAC) of <115 mm to identify severe acute malnutrition (SAM). However, it is currently unclear how MUAC relates to the other indicator used to define acute malnutrition: weight-for-height Z-score (WHZ).

**Methods:**

Secondary data from >11,000 Cambodian children, obtained by different surveys between 2010 and 2012, was used to calculate sensitivity and ROC curves for MUAC and WHZ.

**Findings:**

The secondary analysis showed that using the current WHO cut-off of 115 mm for screening for severe acute malnutrition over 90% of children with a weight-for-height z-score (WHZ) <−3 would have been missed. Reversely, WHZ<−3 missed 80% of the children with a MUAC<115 mm.

**Conclusions:**

The current WHO cut-off for screening for SAM should be changed upwards from the current 115 mm. In the Cambodian data-set, a cut-off of 133 mm would allow inclusion of >65% of children with a WHZ<−3. Importantly, MUAC and WHZ identified different sub-groups of children with acute malnutrition, therefore these 2 indicators should be regarded as independent from each other. We suggest a 2-step model with MUAC used a screening at community level, followed by MUAC and WHZ measured at a primary health care unit, with both indicators used independently to diagnose severe acute malnutrition. Current guidelines should be changed to reflect this, with treatment initiated when either MUAC <115 mm or WHZ<−3.

## Introduction

Acute malnutrition continues to be a major public health concern worldwide. Wasting (Weight-for-Height Z-score (WHZ)<−2) affected over 50 million children under the age of 5 years, or 8% of all under-fives, in 2011 [1]. Severe acute malnutrition (SAM, WHZ<−3) affected approximately 19 million children under the age of five. Acute malnutrition places child at great risk for death, with almost 8% of global child deaths (or over 500,000 deaths per year) directly due to SAM [1]. Community-based treatment, using ready-to-use-therapeutic foods (RUTFs) is appropriate for most children with SAM, as an alternative to in-patient treatment that is required for cases with severe medical complications, and infants below 6 month old. It is estimated that by proper identification and management of acute malnutrition alone, over 400,000 child deaths per year could be prevented [2].

In the latest 2013 guidelines for the management of severe acute malnutrition, the World Health Organization recommends to use Mid Upper Arm Circumference (MUAC) at community level to screen for SAM [3]. A cut-off of <115 mm is currently recommended to be used for all children between 6 and 59 months of age to identify SAM. However, for primary health care facilities, two different indicators are recommended by the World Health Organization to identify SAM: a MUAC<115 mm or a WHZ<−3 [3]. For moderate acute malnutrition (MAM), the recommended cut-offs are between 115 and 125 mm for MUAC or between −3 and −2 for WHZ. Screening for MAM is used when there are programmes that aim to prevent SAM, in addition to programmes for treatment of SAM.

Uncertainty exists however on how MUAC and WHZ relate to each other. Collins et al. recommended use of MUAC at community level [4], as MUAC is considered to reflect mortality risk better than WHZ [5]. Indeed, the World Health Organization recommends MUAC as independent indicator for admission into therapeutic feeding programs [3]. However, the specificity of both MUAC and WHZ for detecting children at risk for death have been reported to be high (>95%) [5]. In contrast, surprisingly low sensitivities (<10%) have been reported for either MUAC<115 mm and WHZ<−3 for predicting death [5], indicating that actually most children at risk for dying are not accurately identified by either of these indicators. Moreover, poor correlations have been reported between MUAC and WHZ-scores [5]–[6]. Although both indicators are measuring nutritional status, it is possible, given the low sensitivity, that these two indicators identify distinctly different sub-sets of children at risk for dying, but fail to cover the complete spectrum of SAM, each missing a considerable proportion of the children with SAM. The current study aimed to identify which indicator is best suited to identify children with acute malnutrition.

## Methods and Data Collected

Cambodia ranks amongst the 42 countries with the highest prevalence of wasting worldwide, with wasting implicated in more than 6,400 child deaths annually [7]. To advise the Ministry of Health of Cambodia on the most effective strategy to identify children with SAM, we assessed the sensitivity and specificity of MUAC on detecting children with a WHZ<−3, using data from 4 recent surveys conducted in Cambodia between 2011 and 2013. Though other cut-offs for MUAC such as <125 mm and <135 mm have been suggested to detect SAM [8], these alternative cut-offs have not been validated at a sufficiently large scale in the literature. Therefore, our objective was to determine the optimal cut-off for MUAC to detect children with either SAM or MAM, considering that even though a MUAC cut-off of 115 mm alone is currently the criteria recommended by WHO to identify SAM at community level, WHZ<−3 is still considered the golden standard to identify SAM in children under 5 years of age.

Data on weight, height, MUAC and age were available for a total of 11,818 children, from 4 different surveys conducted by several organizations (UNICEF, WHO, WFP, World Vision and International Relief and Development) between 2011 and 2013. The target population was children aged 0–59 months. Measurements of MUAC (to the nearest 1 mm) were made using a non-stretch tape measure (provided through UNICEF). Weight was measured with the child wearing no clothes, or only light clothes, and was recorded to the nearest 0.1 kg by a Salter scales. Length/height was measured to the nearest 0.1 cm with a “Holtain infantometer”. Age was determined (calculated in the nearest months) by asking of both the child's age and date of birth.

All data were entered into PASW statistics 18 (Chicago, USA). Anthropometric data was transformed to Z-scores using WHO Anthro (version 3.2.2, January 2011) software application that provides global references for child growth and development. The sensitivity, specificity, False Positive Rate values of the MUAC were determined using WHZ as gold standard. Kappa statistic (K) were calculated as test of association and reproducibility was considered excellent at K>75%, good at 40%≥K≥75% or marginal at K<40% [9]. Receiver–operator characteristic (ROC) curves were constructed to present the relationship of MUAC with WHZ for different cut-offs.

## Results

### Comparison MUAC with WHZ

From the total of 11,818 children for whom data was available, 51·0% were male. Mean age was 26.3±13.9 months (45.7% of children were less than 2 years old, 30.9% between 2 and 3 years old and the remainder between 3 and 5 years old). Mean MUAC was 144±11 mm and ranged between 77 mm to 194 mm. The prevalence of SAM as indicated by MUAC<115 mm was only 0.4% (95% CI: 0.31–0.54). The prevalence of MAM as indicated by MUAC between 115 mm and 125 mm was 2.9% (95% CI: 2.62–3.23). In comparison, the prevalence of SAM and MAM as indicated by WHZ was much higher, at 1.4% (95% CI: 1.18–1.61) and 9.2% (95% CI: 8.68–9.74) respectively.

The current cut-offs of MUAC for diagnosing acute malnutrition (MUAC<125 mm), MAM (115 mm≤MUAC<125 mm) and SAM (MUAC<115 mm) compared poorly to the same categories as defined by WHZ, with Kappa's of 21·3%, 16·7% and 8·6% respectively (Table 1–3).

**Table 1:** Validity of MUAC for wasting (WHZ<−2).

MUAC<12.5	WHZ<−2		Total
	Positive	Negative	
Positive	209	187	396
Negative	1,045	10,377	11,422
Total	1,254	10,564	**11,818**

Note: Sensitivity  = 16.7 and specificity  = 98.2 and Kappa = 21.3%.

**Table 2:** Validity of MUAC for MAM (−3<WHZ<−2).

11.5≤MUAC<12.5	−3≤WHZ <−2		Total
	Positive	Negative	
Positive	146	200	346
Negative	943	10,529	11,472
Total	1,089	10,729	**11,818**

Note: Sensitivity = 13.4 and specificity  = 98.1 and Kappa = 16.7%.

**Table 3:** Validity of MUAC for SAM (WHZ<−3).

MUAC<11.5	WHZ<−3		Total
	Positive	Negative	
Positive	10	40	50
Negative	155	11,613	11,768
Total	165	11,653	**11,818**

Note: Sensitivity  = 6.1 and specificity  = 99.7 and Kappa = 8.6%.

### ROC curves

**Figure 1:**
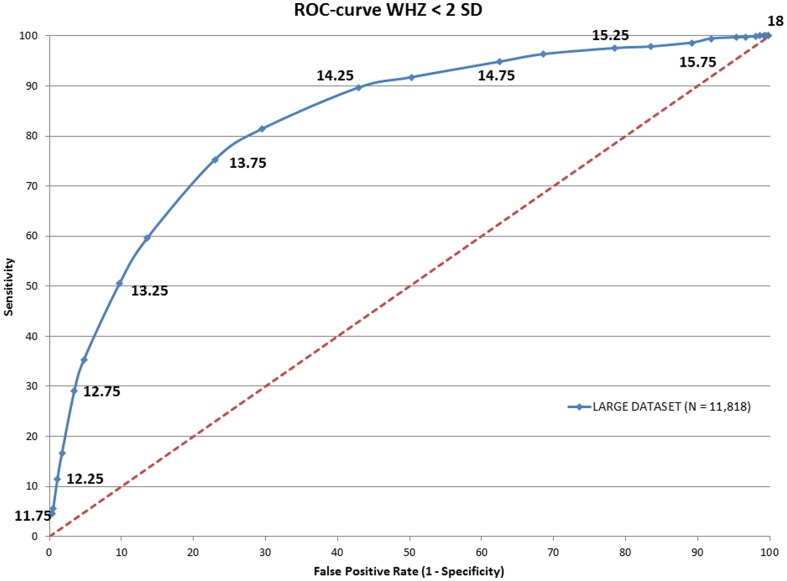
ROC curve of the MUAC score in against WHZ<-2SD.

**Figure 2:**
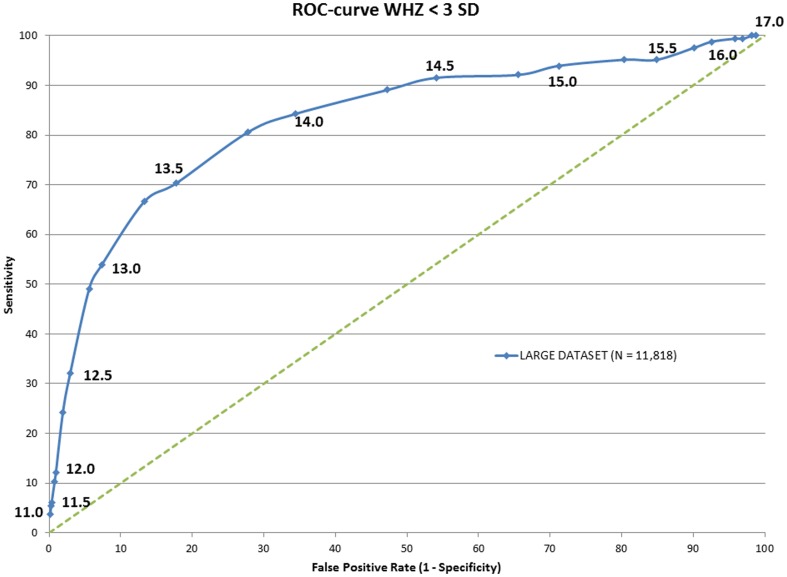
ROC curve of the MUAC score in against WHZ<-3SD.

To determine whether the power of MUAC to predict acute malnutrition (MAM+SAM) and severe acute (SAM) malnutrition could be improved, Receiver-Operator Characteristic (ROC) curves were drawn against WHZ (Fig. 1 and 2 respectively). Areas under curve (AUC) were calculated and showed an optimal MUAC cut off to detect acute malnutrition of 138 mm and to detect SAM of 133 mm (Table 4).

**Table 4:** Areas under curve (AUC) for wasting and severe acute malnutrition.

WHZ<−3SD				WHZ<−2SD			
MUAC cut-off (mm)	false positive rate	Sensitivity	AUC	MUAC cut-off	false positive rate	Sensitivity	AUC
115	0.34	6.06	0.59201	11.5	0.28	1.59	0.53836
							
125	2.94	32.12	0.72159	12.5	1.77	16.67	0.64685
127.5	5.59	49.09	0.78546	12.75	3.48	29.03	0.6991
13	7.37	53.94	0.79601	13	4.79	35.25	0.71973
**132.5**	**13.32**	**66.67**	**0.82071**	13.25	9.74	50.48	0.75829
135	17.74	70.3	0.81128	13.5	13.59	59.57	0.77878
137.5	27.81	80.61	0.81151	**13.75**	**23**	**75.28**	**0.80714**
14	34.37	84.24	0.79763	14	29.56	81.42	0.80644
142.5	47.27	89.09	0.76133	14.25	42.89	89.63	0.78934

## Discussion

MUAC has been considered a valid and simple screening tool to identify SAM in children under 5 years of age. In our data set however, MUAC<115 mm identified only 10 out of 165 children with WHZ<−3. Reversely, WHZ<−3 only identified 10 out of 50 children with a MUAC<115 mm. This means that with the current guidelines of WHO, which were updated in 2013, using only MUAC<115 mm at community level to screen for SAM, over 90% (155 out of 165) of children with a WHZ<−3 are missed and left without treatment. If these findings are representative for other populations, the implementation of the current guidelines on a global scale would mean that of the 19 million children in 2011 with SAM (as defined as a WHZ<−3), 17 million would have been missed by screening with MUAC only. However, changing current guidelines from using MUAC for screening to WHZ only at community level would have resulted in 80% of children with a MUAC<115 mm being missed.

Although we have used data from only Cambodia in the present study, we are confident that our findings are readily applicable to other countries. Fernandez et al, using data from 39 surveys in 10 mostly African countries, showed that a MUAC<135 mm, which is close to our optimal cut off of 133 mm, was optimal to identify SAM (highest AUC in ROC curve), with a sensitivity of 84.5%[8]. Moreover, the WHO recommendations on the diagnosis and management of SAM are applied worldwide, and we have no reasons to assume that Cambodia is different from other countries or represents an extreme population or extraordinary circumstances. Therefore, the findings of this study have direct relevance for the global recommendations on the diagnosis and treatment criteria of acute malnutrition as currently formulated by WHO.

Briend et al showed that both MUAC<115 mm and WHZ<−3 carry a great risk for death [5]. However, at community level, MUAC has many advantages over WHZ. The authors argue that there is no benefit of using WHZ in addition to MUAC as specificity of MUAC is higher than of WHZ to predict subsequent death. But as shown in our data, MUAC<115 mm and WHZ<−3 clearly identify a distinctly different set of children with malnutrition, with hardly any overlap between the 2 indicators. This is actually not surprising, as they each measure different aspects of body composition, reflecting perhaps different categories of malnutrition Therefore, we propose that both indicators should be regarded as independent from each other, and cannot be used as substitutes for each other.

It is important to understand why MUAC and WHZ identify different sub-groups of children at risk of death. Chomtho and colleagues showed that MUAC was strongly related to fat mass in children, but related poorly to fat free mass or overall weight [10]. In contrast, WHZ cannot discriminate between fat and lean body mass, and therefore reflects both fat mass and lean body mass [11]. Indeed, it was recently shown that fat mass might range anywhere from close to 0% to more than 15% in newborn babies with the same weight of 3 kg [12]. Both fat mass and lean body mass have been shown to be important for immune function and survival, but they affect different immunological functions. Fat mass is linked to immune function through leptin. Leptin is produced primarily by adipocytes, and a key regulator of immune responses, with leptin favoring a Th1 immune response profile [13]. Indeed, exogenous leptin strongly improved host defense against for example Steptococcus *pneumoniae* in starved mice [14]. On the other hand, lean body mass is linked to the immune responses through different roles of amino-acids in the immune system, such as the acute phase response. Also, glutamate and the sulfur-containing amino acids are important for anti-oxidant status through glutathione, and arginine is important for NO production [15]. Hence, it is likely that MUAC and WHZ identify distinctly different categories of children at risk to die from different types of infection (viral/bacterial/parasitical), which require different immune responses. Grossly simplified this could mean that a low MUAC score (and a WHZ>−3) might render a child at a high risk to die from infectious diseases which need a typical Th1 response (e.g. viral infections), whereas a low WHZ score might put a child at a higher risk for death by infectious diseases requiring an adequate humoral immune response (e.g. malaria). And changes in types of most prevalent infection pressure due to e.g. seasonality might then result in either MUAC or WHZ being more predictive of risk for death. The idea that body composition is related to differences in infection pressure and survival is not completely new: Wells put forward the hypothesis that differences in body composition, especially in distribution of fat mass, as seen between Asians, Caucasians and Africans might be due to differences in infection pressure, with localization of adipose tissue deposits specifically contributing to survival [16].

### Way Forward

MUAC is clearly an easier, quicker and more robust screening tool at community level than weight-for-height. However, the cut-off of 115 mm is incorrect as single criteria for the diagnosis of malnutrition, as most children with a WHZ<−3 are missed. At the same time, WHZ<−3 is also incorrect as single criteria for the diagnosis of acute malnutrition, as 80% of children with MUAC<115 mm are missed. Yet, the current guidelines from WHO stipulate that health-care workers should use either MUAC *or* WHZ of infants and children who are 6–59 months of age, to commence treatment for SAM. However, by giving health-care workers the choice of either one of the indicators, the group of children identified as malnourished by the other indicator, and who also have a markedly increased risk of death, is almost completely excluded. Therefore, we recommend that MUAC<115 mm should be considered as an independent therapeutic criteria of SAM, measured in parallel and in addition to WHZ. Importantly, children should be diagnosed as SAM and treated with therapeutic feeding when *either* MUAC<115 mm *or* WHZ<−3, regardless of the value of the other indicator, as both groups of children have a high risk of death.

**Figure 3:**
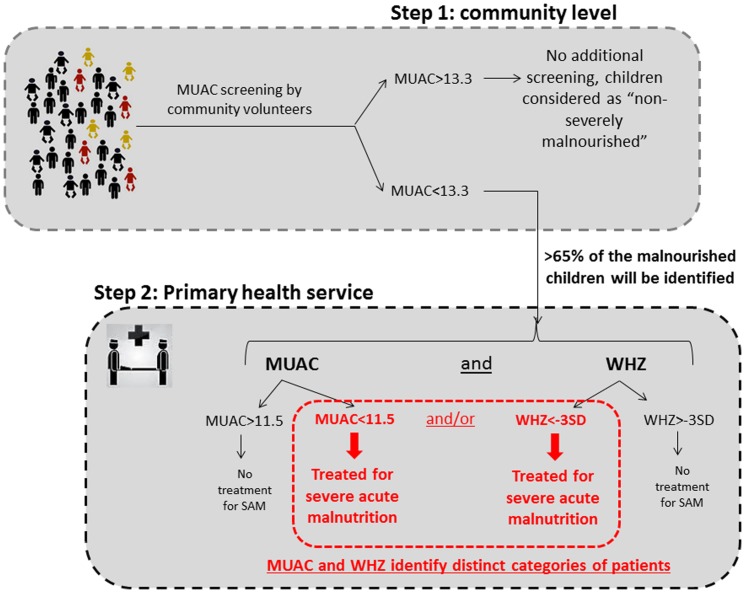
Two-step screening procedure for severe acute malnutrition at community level.

For screening for SAM at community level we propose a two-step screening procedure (figure 3), such as used in many public health programs that aim to identify disease in populations. As a first step, MUAC can be effectively used as a simple and robust screening tool using a broader cutoff of for example <133 mm to ensure inclusion of as many children with SAM as possible. Thus the current cut-off for *screening* for severe acute malnutrition at community level should be changed from 115 mm to 133 mm (*step one, screening cut-off*). This will identify, according to our data set, over 65% of the children with a WHZ<−3. Then, as a *second step*, all children with a MUAC below the screening cut-off (e.g. <133 mm) should be assessed at a secondary facility, e.g. a primary health care center, for weight, height *and* MUAC measurements. Subsequently, malnutrition can be diagnosed as SAM using both MUAC (now at diagnostic cut-off: <115 mm) and WHZ (<−3) criteria independently of each other. Such an approach will not only solve the current confusion and conflicting findings, but also improve the cost-effectiveness of screening programs and the treatment because sensitivity will be significantly improved.

**Figure 4:**
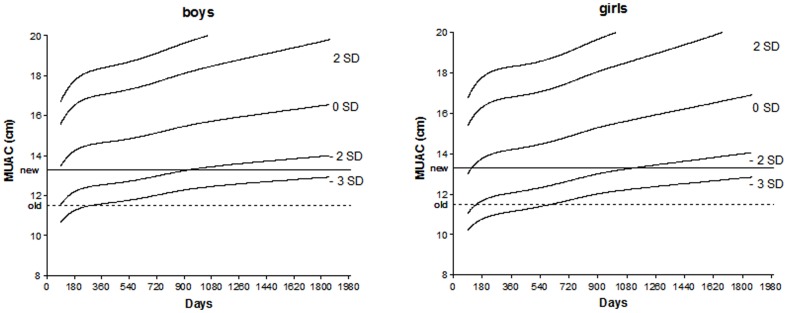
MUAC-for-Age Z-scores according to the gender (boys and girl) from WHO anthropometric database in comparison to a single cut-off of 13.3 cm.

Although in this Cambodian data set, a MUAC of 133 mm was optimal, a wider consensus should be reached on a global, ideal cut-off, taking not only feasibility into account, but also specificity. Having a high number of false-positive cases, could lead to many children visiting health center being turned away without intervention, undermining confidence in the health system. Moreover, MUAC was highly dependent on age in our dataset, and increased especially after 2 years of age (data not shown). Hence, age-specific cut-offs for MUAC might have a higher sensitivity than a single cut-off, although this could complicate community-level screening. But as clearly shown in Figure 4, as single cut-off for MUAC in children between 6 and 59 months would tend to overestimate the prevalence of malnutrition in younger children, especially younger girls, and underestimate the prevalence of malnutrition in older children, especially boys.

For countries with a policy for screening and treating of also moderate acute malnutrition, the same rationale prevails. A similar two-step screening procedure should be applied, with community screening using a MUAC<138 mm (optimal cut-off in Cambodain data set), and consequent secondary diagnostic follow-up at a primary health center using a treatment criteria of MUAC<125 mm or WHZ<−2. Community screening by MUAC<138 mm will, according to our data set, identify over 75% of the children with a WHZ<−2. Indeed, as over 80% of children with WHZ<−3 are identified by a MUAC<138 mm, it could be argued that for simplicity, one cut-off for community screening for wasting (combining MAM and SAM), using MUAC<138 mm would suffice, although this would double the false-positive rate (from 13% to 28%).

As highlighted in the updated WHO guidelines for management of SAM [3], several research priorities remain, including refinement of the current cut-off values for MUAC for children at different age ranges, such as those below 6 months and >5 years of age. In addition, data are lacking on how these different anthropometric indices predict disease-specific morbidity and mortality. These data could allow establishment of optimal cut-offs based on regional or country characteristics, such as baseline mortality rates and infection pressure. Regardless of this current lack of data, screening for severe acute malnutrition can be improved today by increasing the cut-off for MUAC.
